# The Human Manufactory

**Published:** 1859-07

**Authors:** 


### THE HUMAN MANUFACTORY.

A man man may eat and drink heartily all day, and sit and lounge about, "doing nothing," in one sense of the word, but his body must keep hard at work all the time, or it will die. Suppose the stomach refuses to work within ten minutes after a hearty dinner, the man would die in convulsions in a few hours, or cholera or cramp colic would rack and wreck him. Suppose the "pores" of the skin, meaning thereby the glandular apparatus with which they are connected, should go on a "strike," we would in an hour be burning up with fever, or "oppression" would weigh down the system, and soon become insupportable. Suppose the liver became "mulish," appetite would be annihilated, food would be loathed, torturing pains would invade the "small of the back," and the head would ache to "bursting." Suppose the kidneys "shut up shop," and dangers more imminent, sufferings more unbearable, and death more certain would be the speedy and inevitable results. If the little workshops of the eye should "close," in an hour we could not shut or open them without physical force, and in another we would be blind; or of the tongue, and it would become as dry as a bone and as stiff as steel. To keep such a complication of machineries in working order for a life time, is a miracle of wisdom, but to "work them" by the pleasures of eating and drinking, is a miracle of benificence.

